# Two Single Nucleotide Polymorphisms (rs2431697 and rs2910164) of miR-146a Are Associated with Risk of Coronary Artery Disease

**DOI:** 10.3390/ijerph14050514

**Published:** 2017-05-10

**Authors:** Yaqin Wang, Xintong Wang, Zhenyu Li, Lulu Chen, Luping Zhou, Chaopeng Li, Dong-sheng Ouyang

**Affiliations:** 1 Department of Clinical Pharmacology, Xiangya Hospital, Central South University, Changsha 410008, China; kfwangyaqin1990@163.com (Y.W.); xintongw0126@163.com (X.W.); zndxchenll@163.com (L.C.); zhouluping6060@163.com (L.Z.); samuro@sina.com (C.L.); 2 Institute of Clinical Pharmacology, Hunan Key Laboratory of Pharmacogenetics, Central South University, Changsha 410078, China; 3 Department of Cardiology, Xiangya Hospital, Central South University, Changsha 410008, China; xintongw0126@163.com

**Keywords:** microRNA-146a, coronary artery disease, gene polymorphism

## Abstract

The coronary artery disease (CAD) is one of the most severe cardiovascular diseases. MicroRNA-146a (miR-146a) influences the pathology of cardiovascular diseases. Two single nucleotide polymorphisms (SNPs) of miR-146a (rs2431697 and rs2910164) have been reported to alter the function or expression of microRNA. The purpose of this study is to evaluate the association between miR-146a gene polymorphism and the risk of CAD in the Chinese population. A total of 353 CAD patients and 368 controls were recruited, and SNPs were analyzed by the matrix-assisted laser desorption/ionization time-of-flight mass spectrometry and Sequenom MassARRAY system. The gene frequencies of rs2431697 and rs2910164 were significantly different between the two groups. The mutant type (T allele) of rs2431697 and wild type (C allele) of rs2910164 were more frequent in CAD patients. T allele carriers in rs2431697 had an increased CAD risk, while G allele of rs2910164 decreased the risk of CAD significantly. In conclusion, we found that the T allele of rs2431697 was a risk factor of CAD in the Chinese population. Meanwhile, we demonstrated that the G allele of rs2910164 decreased the susceptibility of CAD.

## 1. Introduction

Coronary artery disease (CAD) is one of the most severe cardiovascular diseases. CAD is caused by the formation of plaques in coronary arteries, and arteries in the heart are thus blocked [1]. Environmental and genetic factors lead to the development and progression of CAD together. One epidemiological study has identified many risk factors leading to CAD including hypertension, diabetes mellitus, smoking, family history, and sedentary lifestyle [2]. However, those risk factors only account for a part of the etiology of CAD, indicating the effect of genetic factors on the variation of CAD [3].

MicroRNAs (miRNAs) are a class of 21- to 24-nucleotide noncoding RNA gene products. They bind to cis-acting regulatory elements of the 3′untranslated region (UTR) of target genes, cause the inhibition of translation, and finally disrupt the expression of the target protein [4]. Much evidence has indicated that miRNAs regulate the progression of atherosclerosis by controlling the function of main cellular participants including endothelial cells, smooth muscle cells, and macrophages [5]. MicroRNA-146a (miR-146a), located at the human chromosome 5q33.3, has been reported to be a regulator of the inflammatory process and influence the pathology of CAD [6].

rs2431697 (C > T) was firstly identified by a genome-wide association study (GWAS) in patients with systemic lupus erythematosus (SLE) [7]. This SNP is located between miR-146a and pituitary tumor-transforming gene 1 (PTTG1). Gene expression analysis revealed that rs2431697 had an effect on the expression level of miR-146a, but not PTTG1 [8]. Several studies explored the association between rs2431697 and SLE [9,10], but the effect of this SNP on CAD has not been reported until now. rs2910164 (C > G) is another common mutation site of miR-146a, and it may alter the function and expression of miRNA [11]. The relationship between rs2910164 and CAD has been studied widely [11,12,13], but results were inconsistent. The purpose of this study is to evaluate the association between two SNPs (rs2431697 and rs2910164) of miR-146a and the risk of CAD in the Chinese population.

## 2. Materials and Methods

### 2.1. Study Population

This study contained 353 CAD patients and 368 controls without CAD. The subjects were recruited from the Xiangya Hospital and the third Xiangya Hospital of Central South University between May and September 2016. The diagnosis of CAD was confirmed by coronary angiography (CA). The diagnostic criteria is at least one major epicardial coronary artery shows more than 50% organic stenosis [14]. The controls were defined to be free of CAD by CA, and assistant diagnoses such as electrocardiograms, clinical examinations, and medical histories were also performed. Subjects with congestive heart failure, rheumatic heart disease, pulmonary heart disease, peripheral vascular disease, chronic kidney disease, or other serious diseases were excluded from this study. Smoking and diseases of hypertension and diabetes mellitus were diagnosed based on other reports [15,16]. The clinical characteristics such as age, sex, height, weight, disease history, family history, and biochemical indexes including triglyceride (TG), total cholesterol (TC), low-density lipoprotein cholesterol (LDL-C), high-density lipoprotein cholesterol (HDL-C), and fasting blood-glucose (FBG) levels were collected. 

The study protocol was approved by Ethics Committee of Xiangya Hospital of Centre South University (registration number: ChiCTR-ROC-16008791). Written informed consent was obtained from each participant.

### 2.2. Sample Collection and SNP Genotyping

Whole blood was collected from each participant and stored at −20 °C. Genomic DNA was extracted from peripheral blood samples with a Qiagen DNA Isolation Kit (Valencia, CA, USA). rs2910164 and rs2431697 were analyzed by the matrix-assisted laser desorption/ionization time-of-flight mass spectrometry (MALDI-TOF MS) and the Sequenom MassARRAY system (Sequenom, San Diego, CA, USA).

### 2.3. Statistical Analysis

Differences in the demographic and clinical characteristics between case and control groups were analyzed by the independent samples *t*-test or the chi-square test (χ^2^ test). Deviations from Hardy-Weinberg equilibrium and the differences in genotype and allele frequencies of rs2431697 and rs2910164 were calculated by the χ^2^ test. The risk factors of CAD and the association between miR-146a gene polymorphism and the severity of CAD were determined by binary logistic analysis. Data was expressed as odds ratios (ORs) and 95% confidence intervals (95% CIs). *p* < 0.05 was considered significant.

## 3. Results

### 3.1. Characteristics of the Study Population

The baseline characteristics of the participants are presented in Table 1. No differences in the distributions of age and gender were found between the control and case groups (*p* > 0.05). Compared with control group, the rate of CAD family history was higher in the case group (*p* = 0.030), and the numbers of smokers and patients with hypertension or diabetes mellitus were more significant (*p* = 0.000; *p* = 0.001; *p* = 0.000). The levels of systolic blood pressure (SBP), diastolic blood pressure (DBP), and FBG in the case group were higher (*p* = 0.020; *p* = 0.000; *p* = 0.015), while TC, HDL-C, and LDL-C levels were significantly lower (*p* = 0.005; *p* = 0.000; *p* = 0.000).

### 3.2. Gene Distribution of rs2431697 and rs2910164

The genotype distribution of rs2431697 and rs2910164 are in agreement with Hardy-Weinberg equilibrium in the case and control groups (case group: χ^2^ = 2.911, *p* = 0.088, control group: χ^2^ = 0.199, *p* = 0.655; case group: χ^2^ = 2.342, *p* = 0.126, control group: χ^2^ = 0.212, *p* = 0.645). 

The genotype and allele frequencies of rs2431697 were significantly different between case and control groups as shown in Table 2 (χ^2^ = 6.118, *p* = 0.047; χ^2^ = 12.236, *p* = 0.002) and the difference was also found in the dominant model (χ^2^ = 3.892, *p* = 0.049). The T allele of rs2431697 is more frequent in case group compared with the control group. 

The distribution frequencies of rs2910164 were significantly different among genotypes, alleles and dominant model (χ^2^ = 8.719, *p* = 0.013; χ^2^ = 17.438, *p* = 0.000; χ^2^ = 8.087, *p* = 0.004) and the G allele was less frequent in the case group.

### 3.3. The Risk Factors of CAD

Logistic regression analysis was performed to determine the risk predictors of CAD. Table 3 showed that smoking, family history of CAD, hypertension, and diabetes mellitus were the major influencing factors of CAD (*p* = 0.000; *p* = 0.000; *p* = 0.000; *p* = 0.000). T allele carriers in rs2431697 had an increased CAD risk (OR = 1.263, 95% CI = 1.042−1.532, *p* = 0.018), while the G allele of rs2910164 decreased the risk of CAD significantly (OR = 0.736, 95% CI = 0.629−0.861, *p* = 0.000), which is consistent with the gene distribution of two SNPs.

### 3.4. The Association between the Gene Polymorphism of miR-146a and Severity of CAD

The severity of CAD was identified by the number of vessels with significant stenosis. Patients with CAD were divided into two groups and were, respectively, 1- and 2-vessels and 3-vessels. Table 4 shows the association between rs2431697, rs2910164, and the severity of CAD. The genotype and allele frequencies of rs2431697 and rs2910164 were almost similar among the different groups (*p*_a_ > 0.05). We failed to find an association between rs2431697, rs2910164, and the severity of CAD (*p*_b_ > 0.05).

## 4. Discussion

We genotyped two SNPs of miR-146a and evaluated the association between those SNPs and CAD in the Chinese population. We found that the gene distribution of rs2431697 and rs2910164 was significantly different between CAD and control subjects. T allele carriers in rs2431697 had an increased CAD risk and the G allele of rs2910164 decreased the risk of CAD. However, we failed to find the effect of two SNPs on the severity of CAD.

miR-146a acted as a regulatory in CAD patients by affecting the expression of interleukin-1 receptor-associated kinase 1 (IRAK1) and tumor necrosis factor receptor associated factor 6 (TRAF6), which were involved in the TLR and IL-1 receptor (IL-1R) signaling pathway, and CAD patients exhibited an increased expression level of miR-146a [17], indicating its effect on CAD. The gene polymorphism affecting miRNA expression may represent an important risk determinant in disease susceptibility. Our study found that the T allele of rs2431697 increased the risk of CAD and that might be because the T allele promoted the expression of miR-146a. The allele frequencies of rs2431697 in our study, respectively, are 19.6% and 80.4%, which is consistent with another study (16.5% and 83.5%) conducted on the Chinese population [9]. GWAS and meta-analysis studies showed that the T allele of rs2431697 increased the risk of SLE by downregulating the expression of miR-146a because the expression of miR-146a is lower in SLE patients [7,9,10,18]. Another study found no association between rs2431697 and the miR-146a level [19]. Those results were inconsistent with ours. Therefore, the effect of rs2431697 on the expression of miR-146a is ambiguous, and further study is needed to confirm the function of this SNP.

rs2910164, located at the precursor of miR-146a, leads to a low production of mature miR-146a [17,20]. This SNP was studied widely, and many researchers have shown that the G allele of rs2910164 decreased the risk of CAD by downregulating the expression of miR-146a [11,12,21,22]. Excitingly, we got the same results and the G allele frequency (43.4%) in our study was similar to other research [12]. Therefore, we can assume that our sampling data is representative, and results are reliable since we duplicate the results of others. However, two researchers failed to find the association between rs2910164 and CAD [13,23]. The conflicts among those studies are due to differences in sample size, experimental bias, inclusion and exclusion criteria, and, possibly, ethnic groups. 

The levels of total cholesterol and low-density lipoprotein cholesterol were significantly higher in the control group than with CAD patients. However, they have no clinical significance since they almost remain within the normal range (TC: 2.8–5.17 mmol/L, LDL-C: 0–3.1 mmol/L). Many researchers have investigated the association between the gene polymorphism of candidate genes and the severity of CAD that was defined by the number of vessels with significant stenosis [14,24]. No association was found between two SNPs of miR-146a and the severity of CAD in our study. We may think therefore that there is no effect of rs2910164 and rs2431697 on the progression of CAD. 

There are some limitations in our study. Firstly, the sample size is not large enough in our study, resulting in insufficient statistical powers for rs2910164 and rs2431697 (0.674 and 0.317). However, the allele frequencies in our study are similar to other Chinese population-based results. Secondly, the subjects were selected from only one area and two centers that may not represent the general population. Thirdly, we attained samples during the summer season and did not consider the effect of the environmental factor on CAD. Therefore, we will enlarge a sample size by recruiting subjects from multicenter studies, multiple regions, and multiple seasons to certify our results in future.

## 5. Conclusions

In conclusion, we found that the T allele of rs2431697 was a risk factor of CAD in the Chinese population. Meanwhile, we demonstrated that the G allele of rs2910164 decreased the susceptibility of CAD.

## Figures and Tables

**Table 1:** Demographic and clinical characteristics of subjects in case and control groups.

Characteristic	Case	Control	*p*
Sample size	353	368	
Age (year)	58 ± 9	57 ± 8	0.056
Gender (male/female)	258/95	258/110	0.376
Smokers (%)	193 (54.67)	126 (34.24)	0.000
Drinking (%)	128 (36.26)	147 (39.95)	0.392
Family history of CAD	42 (11.90)	24 (6.52)	0.030
Hypertension	233 (66.01)	197 (53.53)	0.001
Diabetes mellitus	107 (30.31)	37 (10.05)	0.000
BMI (kg/m^2^)	24.86 ± 2.98	25.33 ± 2.97	0.071
SBP (mm Hg)	135.86 ± 17.19	132.52 ± 20.98	0.020
DBP (mm Hg)	83.40 ± 10.84	79.21 ± 12.23	0.000
FBG (mmol/L)	6.25 ± 2.57	5.83 ± 1.49	0.015
TC (mmol/L)	4.88 ± 2.01	5.22 ± 1.05	0.005
TG (mmol/L)	1.96 ± 1.50	1.93 ± 1.24	0.742
HDL-C (mmol/L)	1.12 ± 0.36	1.25 ± 0.27	0.000
LDL-C (mmol/L)	2.69 ± 0.88	3.07 ± 0.91	0.000

CAD: coronary artery disease; BMI: body mass index, calculated by weigh/(height)^2^; SBP: systolic blood pressure; DBP: diastolic blood pressure; FBG: fasting blood glucose; TC: total cholesterol; TG: triglycerides; HDL-C: high-density lipoprotein cholesterol; LDL-C: low-density lipoprotein cholesterol.

**Table 2:** Gene distribution of rs2431697 and rs2910164 in the case and control groups.

Variable		Case	Control	χ^2^	*p*
**rs2431697**					
**Genotype**	CC	219 (62.0)	254 (69.0)	6.118	0.047
	CT	111 (31.4)	102 (27.7)		
	TT	23 (6.6)	12 (3.3)		
**Allele**	C	549 (77.8)	610 (82.9)	12.236	0.002
	T	157 (22.2)	126(17.1)		
**Dominant model**	CC	219 (62.0)	254 (69.0)	3.892	0.049
	CT + TT	134 (38.0)	114 (31.0)		
**Recessive model**	CC + CT	330 (93.4)	356 (96.7)	4.138	0.126
	TT	23 (6.6)	12 (3.3)		
**rs2910164**					
**Genotype**	CC	136 (38.5)	105 (28.5)	8.719	0.013
	CG	155 (43.9)	179 (48.7)		
	GG	62 (17.6)	84 (22.8)		
**Allele**	C	427 (60.5)	389 (52.9)	17.438	0.000
	G	279 (39.5)	347 (47.1)		
**Dominant model**	CC	136 (38.5)	105 (28.5)	8.087	0.004
	CG + GG	217 (61.5)	263 (71.5)		
**Recessive model**	CC + CG	291 (82.4)	284 (77.2)	3.090	0.079
	GG	62 (17.6)	84 (22.8)		

**Table 3:** Logistic regression analysis of the risk in patients with CAD.

Variable	OR (95% CI)	*p*
Smokers	1.719 (1.378−2.144)	0.000
Family history of CAD	1.865 (1.265−2.750)	0.000
Hypertension	1.632 (1.299−2.049)	0.000
Diabetes mellitus	3.749 (2.786−5.045)	0.000
rs2431697	1.263 (1.042−1.532)	0.018
rs2910164	0.736 (0.629−0.861)	0.000

CAD: coronary artery disease.

**Table 4:** The association between rs2910164 and rs2431697 and the severity of CAD.

Variable		Number of Vessels		χ^2^	*p*_a_	OR (95% CI)	*p*_b_
		1 and 2 (*n* = 194)	3 (*n* = 159)				
rs2431697							
Genotype	CC	120 (61.9)	99 (62.3)	0.111	0.946	1.009 (0.717–1.419)	0.961
	CT	62 (31.9)	49 (30.8)				
	TT	12 (6.2)	11 (6.9)				
Allele	C	302 (77.8)	247 (77.7)	0.221	0.895	1.009 (0.792–1.284)	0.944
	T	86 (22.2)	71 (22.3)				
rs2910164							
Genotype	CC	70 (36.1)	66 (41.5)	1.103	0.576	0.882 (0.658−1.182)	0.399
	CG	89 (45.9)	66 (41.5)				
	GG	35 (18.0)	27 (17.0)				
Allele	C	229 (59.0)	198 (62.3)	2.207	0.332	0.882 (0.717−1.084)	0.233
	G	159 (41.0)	120 (37.7)				

CAD: coronary artery disease. *p*_a_ values are obtained from χ^2^ test. *p*_b_ values are obtained from binary logistic analysis.
